# Bilateral Giant Supraclinoid Carotid Aneurysms Causing Obstructive Hydrocephalus: Case Report and Literature Review

**DOI:** 10.7759/cureus.57780

**Published:** 2024-04-07

**Authors:** Dawoud I Khattab, Baha Aljeradat, Dima R Batarseh, Husesein Al-Abadi, Mahmoud Shehadeh

**Affiliations:** 1 Neurological Surgery, Al-Basheer Government Hospital, Amman, JOR; 2 Neurological Surgery, Jordan University Hospital, Amman, JOR

**Keywords:** supraclinoid carotid artery aneurysm, internal carotid artery aneurysm, bilateral intracranial aneurysm, hydrocephlus, giant intracranial aneurysm

## Abstract

Intracranial aneurysms are common conditions that are usually asymptomatic and found incidentally, yet they can rupture and lead to subarachnoid hemorrhage, in addition to causing mass effects, especially with larger aneurysms. Bilateral supraclinoid aneurysms are extremely rare and were reported in only two instances. These aneurysms can cause a range of symptoms and require careful management. We present the case of a 42-year-old man with no concomitant medical conditions who presented with a persistent headache and deteriorating visual acuity over time. Neurological examination was unremarkable. Connective tissue diseases were ruled out by clinical and laboratory testing. Bilateral large, partly thrombosed supraclinoid segment fusiform aneurysms of the internal carotid artery that are causing midbrain compression and obstructive hydrocephalus were shown by brain CT, CT angiography, MRI, and MR angiography (MRA). Both surgery and endovascular treatment were denied by the patient. However, a ventriculoperitoneal shunt was placed in an outside center and relieved the patient's symptoms. The patient is being followed up. In conclusion, bilateral giant aneurysms of the internal carotid artery present unique challenges and can lead to various clinical manifestations and effects on surrounding structures. In this case, we reported the first instance of obstructed hydrocephalus caused by the largest bilateral supraclinoid carotid aneurysms.

## Introduction

Intracranial aneurysms are considered the leading cause of subarachnoid hemorrhage (SAH), with an estimated incidence of six to 16 cases per 100,000 individuals per year [1].

Among patients diagnosed with unruptured intracranial lesions, it is found that approximately 20% of them will have multiple aneurysms [2]. Individuals who have multiple aneurysms experience higher mortality rates and significantly worse outcomes compared to those with only a single aneurysm [3].

Giant cerebral aneurysms, defined as aneurysms with a diameter exceeding 25 mm, are less common, yet constitute a relatively significant proportion of certain locations like intracavernous aneurysms [4].

Supraclenoid aneurysms are intradural aneurysms that originate from the internal carotid artery (ICA) distal to the distal dural ring, to the carotid terminus. The description of bilateral giant supraclinoid aneurysms is extremely rare [5].

We describe the largest bilateral supraclinoid internal carotid aneurysms and the first case of the largest bilateral supraclinoid internal carotid aneurysms presenting with hydrocephalus

This case report follows the CARE Guidelines [6].

## Case presentation

A 42-year-old Middle Eastern male patient with no comorbidities was transferred from a primary medical center in a refugee camp to our neurological surgery clinic with a complaint of chronic headache that was associated with progressive loss of visual acuity for 2 years. The headache partially responded to simple analgesia like acetaminophen and ibuprofen and was pulsatile in nature.

The patient had no focal neurological deficits. Connective tissue disorders were ruled out, and lab tests including complete blood count, kidney function test, liver panel, and coagulation profile were ordered and were within normal limits. Funduscopic Examination showed a hyperemic optic disc.

**Figure 1:**
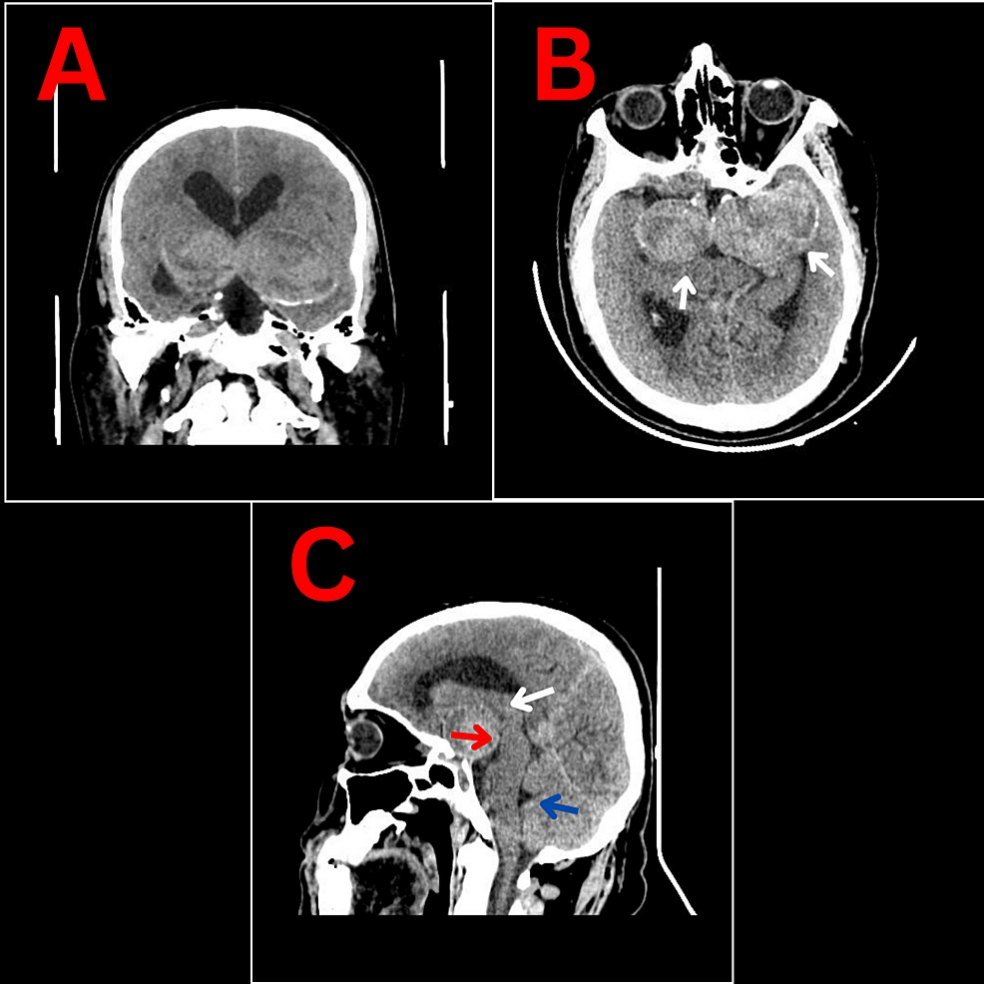
Head CT Scan without contrast showing the bilateral huge supraclinoid carotid aneurysms. A: Coronal cut. B: Axial cut showing the giant bilateral partially thrombosed supraclinoid carotid aneurism holding calcification (arrow) surrounded by perilesional edema causing compression on midbrain and hydrocephalus by obstructing the third ventricle. C: Sagittal view showing the aneurysms compressing the thalamic and hypothalamic structures (white arrow), as well as the superior anterior aspect of the brainstem (red arrow), the 4th ventricle not dilated (blue arrow).

**Figure 2:**
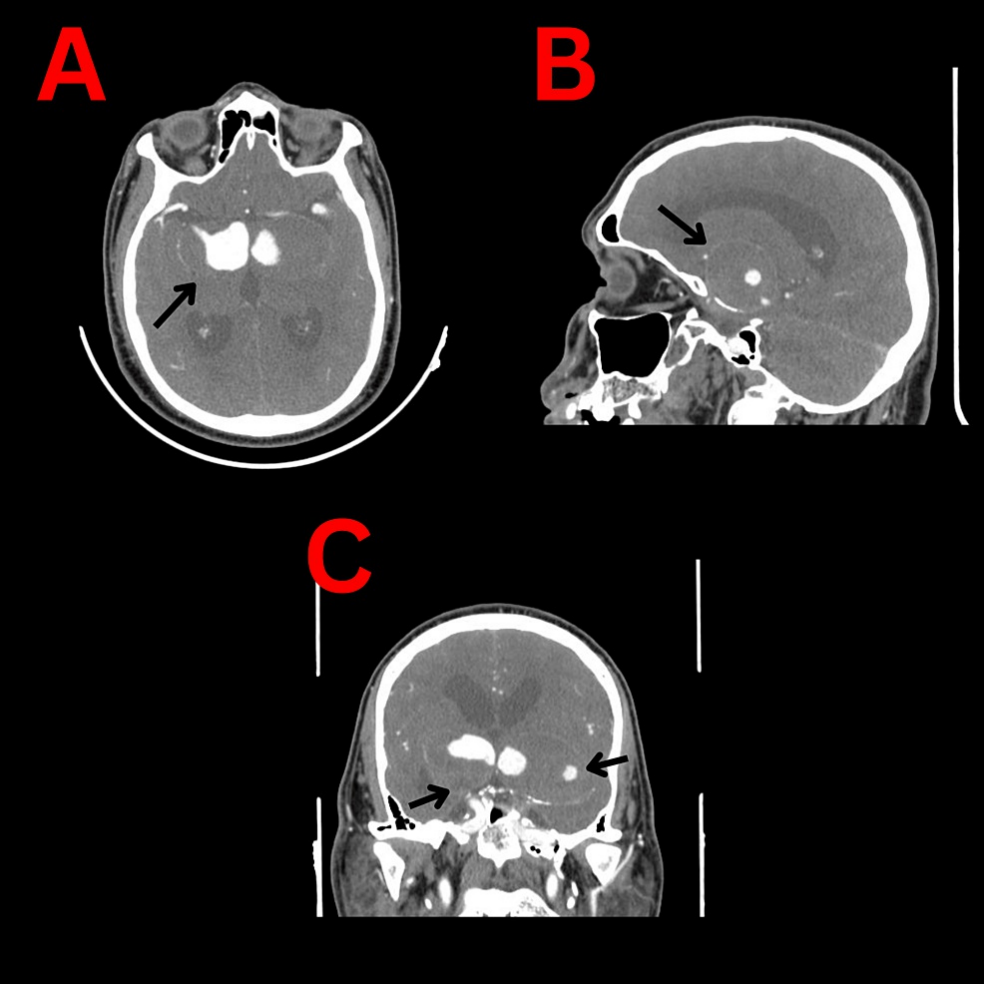
CT angiogram showing the bilateral huge aneurysm (the black arrows) A: Axial cut showing bilateral giant aneurysms of the internal carotid arteries . B: Sagittal view showing the aneurysm. C: Coronal CT angiogram cut demonstrating the bilateral partially thrombosed aneurysms.

Computed tomography (CT) of the brain (Figure 1) and CT angiogram (CTA) (Figure 2) showed bilateral giant, partially thrombosed supraclinoid aneurysms of the internal carotid artery (ICA) causing compression on the midbrain, more so on the left than on the right and resulting in obstructed hydrocephalus.

**Figure 3:**
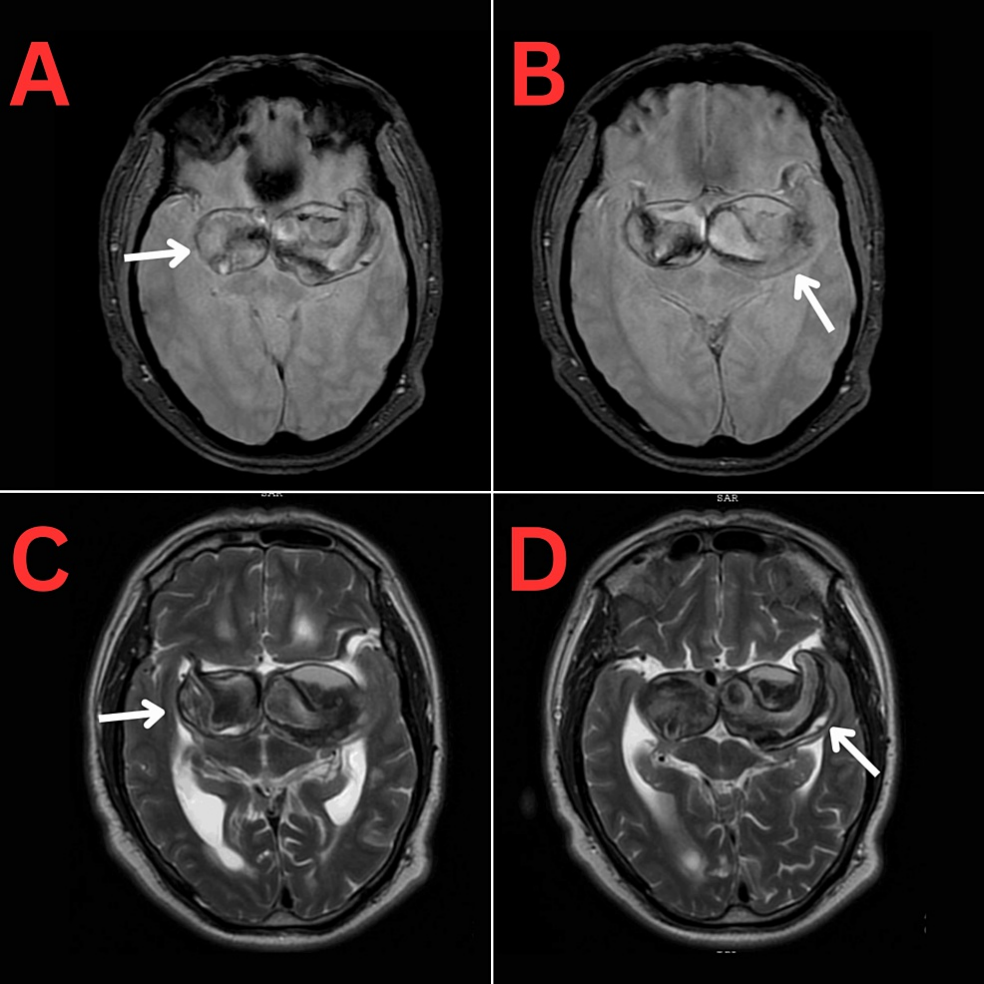
Axial T2-weighted gradient echo (GRE) MRI (A and B) and T2-weighted MRI (C and D) showing the bilateral giant supraclinoid carotid aneurysms (white arrows).

**Figure 4:**
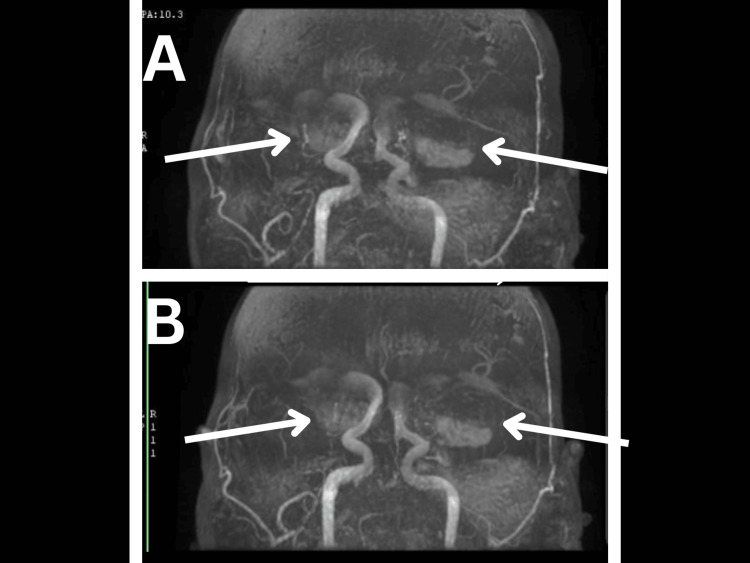
MR angiography showing the bilateral aneurysms (arrows) originating from the supraclinoid segment of the ICAs.

An MRI (shown in Figure 3) was done 2 weeks later and confirmed the presence of the bilateral partially thrombosed aneurysms and showed compression on the adjacent brain parenchyma, midbrain, and Sylvian aqueduct resulting in a moderate degree of obstructive hydrocephalus with periventricular seepage. MR angiography (MRA) (Figure 4) has shown the aneurysms to be originating from the supraclinoid segments of both ICAs and measuring 5.1*3.8*3.7 cm for the left aneurysm and 2.7*2.9*3 cm for the right aneurysm. 

A ventriculoperitoneal shunt was inserted outside our center, which improved the patient’s symptoms. However, the patient refused surgical or endovascular management, partially due to his situation in a refugee camp, and is being followed up currently.

## Discussion

Intracranial aneurysms can be found in up to 3% of the general population, with the majority of them being asymptomatic. However, such aneurysms can rupture and thus lead to subarachnoid hemorrhage and cause other clinical manifestations in many instances [7,8].

Bilateral supraclinoid carotid artery aneurysms are rare with not so many reports in the literature and reports on giant ones are even more rare. Upon reviewing the literature, we only identified two reports of a bilateral supraclinoid carotid artery aneurysm [9,10]. Both cases are presented in (Table 1). 

**Table 1:** Literature review findings of bilateral supraclinoid ICA aneurysms. F - Female, NA - Not Available or Not Applicable, CT - Computed Tomography, MRA - Magnetic Resonance Angiography, ICA - Internal Carotid Artery, cm - Centimeters, mm - Millimeters, DSA - Digital Subtraction Angiography, TOF - Time-Of-Flight (MRI sequence), Rt-Right, Lt-Left, F/U - follow up.

Authors and citation	Age	gender	clinical presentation	examination	labs	Associated dieases	MRI	DSA	max size	treatment	outcome	F/U
Badrawi N, Iqbal SS, Ahmed A, Iqbal SS. [9]	44	F	Chronic headache associated with progressive loss of visual acuity for 6 months. The headache was unilateral to the Lt side and throbbing in nature. The headache was associated with blurring of vision, photophobia.	Reduced visual acuity.	N/A		Bilateral supraclinoid ICA saccular aneurysms were identified. The Rt aneurysm abutted the Rt optic nerve after the ophthalmic artery's origin, while the Lt aneurysm compressed the Lt optic nerve and optic chiasm near the Lt ophthalmic artery's origin. TOF images illustrated these aneurysms.	Wide-neck aneurysm in the supraclinoid segment of the right internal carotid artery measuring 6 mm in diameter. And another wide-neck, lobulated aneurysm was found in the paraophthalmic segment of the left internal carotid artery, measuring 8 mm in diameter.	6 mm on the Rt. 8 mm on the Lt.	Flow-diverter stent insertion	Tolerated the procedure well	3 years
García Carreira MC, Cánovas Vergé D, Marco Igual M, Hervàs Pujol M. [10]	49	F	Progressive loss of visual acuity over 5 years. Also had retro-orbital pain for few weeks.	Bilateral papillary pallor and decreased visual acuity. (Rt Eye: 0.8; Lt Eye: 0.5)	Negative or normal	None	Carotid arteries dilation in the supraclinoid segment, resulting in the compression of the optic nerves. MR angiography showed bilateral aneurysms at the emergence of the right ophthalmic artery ( 16 mm × 12 mm, neck 8.38 mm) and the left ophthalmic artery ( 17 mm, neck 12 mm)		Lt: 17 mm. Rt: 16 mm.			

As Table 1 shows, our case is unique and extremely rare as it represents bilateral supraclinoid aneurysms that are also giant (the largest ones in the literature so far) and are associated with hydrocephalus, which was not reported previously to our knowledge.

About 10% of supraclinoid aneurysms of the carotid artery cause neurological symptoms related to mass effect, including headache, and visual impairment [10]. Both the two previously reported cases and ours presented with headache and loss of visual acuity.

Although it is well known that CT angiography is less sensitive than Digital Subtraction Angiography (DSA), which remains the gold standard, it is generally accepted that the CTA is highly accurate in the diagnosis of intracranial aneurysms. Comparison studies between CTA and DSA for the detection and evaluation of intracranial aneurysms find that CTA is highly sensitive, and specific and can be used as an alternative to DSA [11,12].

DSA couldn’t be done in our case due to financial issues, so we continued with CTA, MRA, and MRI which showed the pre-described findings.

If these aneurysms become symptomatic, it is wise to treat them as they can lead to major neurological deficits. At the same time, the intervention needs to be individualized [13].

The management of intracranial aneurysms can be challenging, especially in similar cases. According to the ISUIA (International Study of Unruptured Intracranial Aneurysms ) study in 2003, the 5-year cumulative rupture rate of aneurysms larger than 25 mm is 6.4% [14]. Others found a 1-year rupture rate of 8.3% [15]. Likewise, risk prediction for bleeding based on the PHASES score is also significant for giant aneurysms in general [16].

Treatment options for intracranial aneurysms include occlusive and reconstructive strategies. Occlusive approaches include surgical or endovascular parent artery ligation. Reconstructive methods involve the application of microsurgical clips, coil embolization with or without a vascular reconstruction device, the use of flow-diverting devices, or the employment of liquid embolic agents [17].

Occlusive strategies can be used if the parent vessel cannot be preserved which can be achieved by Hunterian techniques involving occlusion of the ICA with or without revascularization which can be done by 1) occlusion alone, 2) occlusion with a low-flow bypass (extracranial artery), and 3) occlusion with a high-flow bypass (saphenous vein or radial artery) [17].

However, the intricate anatomy surrounding the paraclinoid ICA can create technical challenges for the microsurgical treatment of aneurysms in this area. This often involves clinoid drilling and difficulty in obtaining proximal control, thereby increasing the risk associated with clipping procedures. Thus, endovascular treatment is becoming more prevalent [5].

The primary goal in treating giant aneurysms is to alleviate mass effect, cure symptoms, and exclude the aneurysm from circulation. This is achieved by reducing transmitted arterial pulsations, promoting clot retraction, and facilitating thrombus resorption, ultimately leading to aneurysm shrinkage and the cessation of episodic growth. The likelihood of benefiting from endovascular treatment is higher in cases with no wall calcification, a shorter duration of neurological symptoms, and successful complete occlusion of the aneurysm lumen [18-20].

In our case, we discussed the case deeply with the patient and his relatives and we informed them about the options of the treatment, but the patient refused definitive management.

## Conclusions

Internal carotid aneurysms can include different portions of the carotid artery, cause different clinical pictures, and lead to different effects on the surrounding structures. Bilateral giant aneurysms can also be associated with mass effects causing unique presentations and requiring individualized management with concern for the aneurysm itself and the nearby structures. We reported the first case of obstructed hydrocephalus caused by bilateral giant supraclinoid carotid aneurysms, in which the management was also hindered by patient-specific factors. Indeed, more work is needed to be able to increase the accessibility of care and to provide management in a timely manner. Further research is necessary to better understand these rare entities.

## References

[1] Broderick JP, Brott TG, Duldner JE, Tomsick T, Leach A (1994). Initial and recurrent bleeding are the major causes of death following subarachnoid hemorrhage. Stroke.

[2] Rinne J, Hernesniemi J, Puranen M, Saari T (1994). Multiple intracranial aneurysms in a defined population: prospective angiographic and clinical study. Neurosurgery.

[3] Andic C, Aydemir F, Kardes O, Gedikoglu M, Akin S (2017). Single-stage endovascular treatment of multiple intracranial aneurysms with combined endovascular techniques: is it safe to treat all at once?. J Neurointerv Surg.

[4] Linskey ME, Sekhar LN, Hirsch W, Yonas H, Horton JA (199026). Aneurysms of the intracavernous carotid artery: clinical presentation, radiographic features, and pathogenesis. Neurosurgery.

[5] Patel BM, Ahmed A, Niemann D (2014). Endovascular treatment of supraclinoid internal carotid artery aneurysms. Neurosurg Clin N Am.

[6] Riley DS, Barber MS, Kienle GS (2017). CARE guidelines for case reports: explanation and elaboration document. J Clin Epidemiol.

[7] Murayama Y, Takao H, Ishibashi T (2016). Risk analysis of unruptured intracranial aneurysms: prospective 10-year cohort study. Stroke.

[8] Williams LN, Brown RD Jr (2013). Management of unruptured intracranial aneurysms. Neurol Clin Pract.

[9] Badrawi N, Iqbal SS, Ahmed A, Iqbal SS (2022). Unruptured bilateral supra-clinoid internal carotid artery aneurysms: a case report. Radiol Case Rep.

[10] García Carreira MC, Cánovas Vergé D, Marco Igual M, Hervàs Pujol M (2013). Bilateral supraclinoid aneurysms associated with progressive visual impairment. Neurología.

[11] Lu L, Zhang LJ, Poon CS (2012). Digital subtraction CT angiography for detection of intracranial aneurysms: comparison with three-dimensional digital subtraction angiography. Radiology.

[12] Wang H, Li W, He H, Luo L, Chen C, Guo Y (2013). 320-detector row CT angiography for detection and evaluation of intracranial aneurysms: comparison with conventional digital subtraction angiography. Clin Radiol.

[13] Morley TP, Barr HW (1969). Giant intracranial aneurysms: diagnosis, course, and management. Clin Neurosurg.

[14] Wiebers DO (2003). Unruptured intracranial aneurysms: natural history, clinical outcome, and risks of surgical and endovascular treatment. Lancet.

[15] Dengler J, Rüfenacht D, Meyer B (2019). Giant intracranial aneurysms: natural history and 1-year case fatality after endovascular or surgical treatment. J Neurosurg.

[16] Greving JP, Wermer MJH, Brown RD (2014). Development of the PHASES score for prediction of risk of rupture of intracranial aneurysms: a pooled analysis of six prospective cohort studies. Lancet Neurol.

[17] Ambekar S, Madhugiri V, Sharma M, Cuellar H, Nanda A (2014). Evolution of management strategies for cavernous carotid aneurysms: a review. World Neurosurg.

[18] Vazquez Añon V, Aymard A, Gobin YP (1992). Balloon occlusion of the internal carotid artery in 40 cases of giant intracavernous aneurysm: technical aspects, cerebral monitoring, and results. Neuroradiology.

[19] Higashida RT, Halbach VV, Dowd C, Barnwell SL, Dormandy B, Bell J, Hieshima GB (1990). Endovascular detachable balloon embolization therapy of cavernous carotid artery aneurysms: results in 87 cases. J Neurosurg.

[20] Cekirge S, Saatci I, Firat MM, Kose G, Belen D, Akalan N, Bertan V (1997). Bilateral cavernous carotid artery aneurysms in a 4-year-old child: endovascular treatment with mechanically detachable coils. Neuroradiology.

